# On the convergence rates of kernel estimator and hazard estimator for widely dependent samples

**DOI:** 10.1186/s13660-018-1659-1

**Published:** 2018-04-03

**Authors:** Yongming Li, Yong Zhou, Chao Liu

**Affiliations:** 1 School of Statistics and Management, Shanghai University of Finance and Economics, Shanghai, China; 2 School of Mathematics and Computer Science, Shangrao Normal University, Shangrao, China

**Keywords:** 62G05, 62G20, Widely orthant dependent, Kernel density estimator, Hazard rate, Strong convergence

## Abstract

In this paper, we establish a Bernstein-type inequality for widely orthant dependent random variables, and obtain the rates of strong convergence for kernel estimators of density and hazard functions, under some suitable conditions.

## Introduction

Let $\{X_{n}, n\geq1\}$ be a sequence of random variables with an unknown marginal probability density function $f(x)$ and distribution function $F(x)$. Assume that $K(x)$ is a known kernel function, the kernel estimate of $f(x)$ and the empirical distribution function of $F(x)$ are given by

1.1 $$ \hat{f}_{n}(x) = \frac{1}{nh_{n}}\sum_{j= 1}^{n} K\biggl(\frac {{x - {X_{j}}}}{{{h_{n}}}}\biggr),\qquad {F_{n}}(x) = n^{-1}\sum _{j= 1}^{n} {I(X_{j}< x)}, $$

where $\{h_{n}, n\geq1\}$ is a sequence of positive bandwidths tending to zero as $n\to\infty$, and $I(\cdot)$ is the indicator of the event specified in the parentheses. Denote the hazard rate of distribution $F(x)$ by $\lambda (t)=f(t)/(1-F(t))$, and it can be estimated by

1.2 $$ \hat{\lambda}_{n}(x) = \frac{\hat{f}_{n}(x)}{{1 - {F_{n}}(x)}}. $$

Works devoted to the estimation of probability density and hazard rate functions include the following. Izenman and Tran ([1], 1990) discussed the uniform consistency and sharp rates of convergence under strong mixing and absolute regularity conditions. Cai ([2], 1998) established the asymptotic normality and the uniform consistency with rates of the kernel estimators for density and hazard functions under a censored dependent model. Liebscher ([3], 2002) derived the rates of uniform strong convergence for density and hazard rate estimators for right censoring based on a stationary strong mixing sequence. Liang et al. ([4], 2005) obtained the optimal convergence rates of the nonlinear wavelet estimators of the hazard rate function when the survival times form a stationary strong mixing sequence. Bouezmarni et al. ([5], 2011) proposed new estimators based on the gamma kernels for density and hazard rate functions which are free of bias, and achieved the optimal rate of convergence in terms of integrated mean squared error, and so on.

On the other hand, different from strong mixing and negatively associated random variables, widely orthant dependence random variables (defined below) were introduced by Wang and Cheng ([6], 2011). Chen et al. ([7], 2016) proved a new type of Nagaev’s inequality and a refined inequality of widely dependent random variables, and as applications, investigated elementary renewal theorems and weighted elementary renewal theorem. Now, let us recall the following definition of widely orthant dependence.

### Definition 1.1

For random sequence $\{X_{n}, n\geq1 \}$, if there exists a finite real sequence $\{g_{U}(n), n\geq1\}$ satisfying, for each $n\geq1$ and for all $x_{i}\in(-\infty, \infty)$, $1\leq i \leq n$,

1.3 $$ P(X_{1}>x_{1}, X_{2}>x_{2}, \ldots, X_{n}>x_{n})\leq g_{U}(n)\prod _{i=1}^{n}P(X_{i}>x_{i}), $$

and there also exists a finite real sequence $\{g_{L}(n), n\geq1\}$ satisfying, for each $n\geq1$ and for all $x_{i}\in(-\infty, \infty)$, $1\leq i \leq n$,

1.4 $$ P(X_{1}\leq x_{1}, X_{2}\leq x_{2}, \ldots, X_{n}\leq x_{n})\leq g_{L}(n)\prod _{i=1}^{n}P(X_{i}\leq x_{i}), $$

then a random sequence $\{X_{n}, n\geq1\}$ is called widely orthant dependent (*WOD*) with dominating coefficients $g(n)=\max\{ g_{U}(n), g_{L}(n)\}$.

Now, we will give two real examples of WOD sequences. The first example of WOD that satisfies the conditions of the main results is given as follows (see Example 1.1) by the framework of Farlie–Gumbel–Morgenstern (FGM) dependence (see Cambanis [8], 1991). The second example (see Example 1.2) does not satisfy the conditions of the paper, but it is useful as an example in the article.

### Example 1.1

A sequence $\{X_{n}, n\geq1\}$ of random variables on $(\Omega, B, P)$ is called FGM if for any $n\in N$ and $(x_{1}, \ldots, x_{n})\in R_{n}$,

1.5 $$ P\{X_{1}\leq x_{1}, \ldots, X_{n}\leq x_{n}\}=\prod_{i=1}^{n} F_{i}(x_{i}) \biggl(1+\sum_{1\leq j< k\leq n}a(j,k) \overline {F}_{j}(x_{j})\overline{F}_{k}(x_{k}) \biggr), $$

the constants $a(\cdot, \cdot)$ are admissible if the $2^{n}$ inequalities $1+\sum_{1\leq j< k\leq n}a(j,k)\varepsilon_{j}\varepsilon _{k}\geq0$ for all $\varepsilon_{j}=-M_{j}$ or $1-m_{j}$ hold, where $M_{j}$ and $m_{j}$ are the supremum and the infimum of the set $\{\{ F_{i}(x), x\in R\} \setminus\{0,1\}\}$. If for some integer *i* the marginal $F_{i}(\cdot)$ is absolutely continuous, then $M_{j}=1$ and $m_{j}=0$, hence $\varepsilon_{i}=\pm1$. Next, by Hashorva and Hüsler ([9], 1999), we have $\sum_{1\leq j< k\leq n}a(j,k)=O(n)$, $n\in N$. Hence, from (1.4) and (1.5), we can take $g_{L}(n)=O (1+\sum_{1\leq j< k\leq n}a(j,k) )=O(n)$, then $P\{X_{1}\leq x_{1}, \ldots, X_{n}\leq x_{n}\}\leq g_{L}(n)\prod_{i=1}^{n} F_{i}(x_{i})$. This implies that the FGM sequence is a WOD sequence, and the conditions of the main results and lemmas are satisfied.

### Example 1.2

Assume that the random vectors $(\xi_{n}, \eta_{n}) $, $n=1,2,\ldots $ , are independent and for each integer $n\geq1$, the random variables $\xi_{n}$ and $\eta_{n}$ are dependent according to the Farlie–Gumbel–Morgenstern copula with the parameter $a_{n}\in[-1,1]$. Suppose that the distributions of $\xi_{n}$ and $\eta_{n}$, $n=1,2, \ldots $ , are absolutely continuous, denoted by $F_{\xi_{n}}$ and $F_{\eta_{n}}$, $n=1,2, \ldots $ , respectively. By Sklar’s theorem (see Chap. 2 of Nelsen RB ([10], 2006)), for each integer $n\geq1$ and any $x_{n}, y_{n}\in(-\infty,+\infty)$, we can construct the cumulative distribution function of $(\xi_{n}, \eta_{n})$ as follows:

$$P(\xi_{n}\leq x_{n}, \eta_{n}\leq y_{n} )=F_{\xi_{n}}(x_{n})F_{\eta_{n}}(y_{n}) \bigl[1+a_{n}\overline{F_{\xi_{n}}}(x_{n}) \overline{F_{\eta_{n}}}(y_{n})\bigr] $$

and

$$P(\xi_{n}> x_{n}, \eta_{n}> y_{n} )= \overline{F_{\xi_{n}}}(x_{n}) \overline {F_{\eta_{n}}}(y_{n}) \bigl[1+a_{n} F_{\xi_{n}}(x_{n})F_{\eta_{n}}(y_{n}) \bigr]. $$

Therefore, for each $n\geq1$, we have

$$\begin{gathered} P(\xi_{n}\leq x_{n}, \eta_{n}\leq y_{n} )\leq2 P(\xi_{n}\leq x_{n}) P(\eta _{n}\leq y_{n} ),\\ P(\xi_{n}> x_{n}, \eta_{n}> y_{n} )\leq2 P(\xi_{n}> x_{n}) P( \eta_{n}> y_{n} ).\end{gathered} $$

Then $\{(\xi_{n}, \eta_{n})\}$ is a sequence of independent bivariate WOD random variables. Thus, for all $x_{j}, y_{j}\in R$,

$$P \Biggl[\bigcap_{j=1}^{n}( \xi_{j}\leq x_{j}, \eta_{j}\leq y_{j} ) \Biggr]\leq 2^{n} \prod_{j=1}^{n} P(\xi_{j}\leq x_{j}) P(\eta_{j}\leq y_{j} ) $$

and

$$P \Biggl[\bigcap_{j=1}^{n}( \xi_{j}> x_{j}, \eta_{j}> y_{j} ) \Biggr]\leq2^{n} \prod_{j=1}^{n} P( \xi_{j}> x_{j}) P(\eta_{j}> y_{j} ). $$

From this, and by Definition 1.1, it is easy to see that the sequence $\{\xi_{1}, \eta_{1}, \ldots, \xi_{n}, \eta_{n}, \ldots\} $ is WOD with $g_{L}(n)=g_{U}(n)=2^{n}$, but the condition of Lemma 2.5 is not satisfied.

We can further refer to some large sample properties of nonparameter estimate based on WOD samples. For instance, Wang et al. ([11], 2013) studied the strong consistency of estimator of fixed design regression model for WOD samples. Shi and Wu ([12], 2014) discussed the strong consistency of kernel density estimator for identically distributed WOD samples. Li et al. ([13], 2015) studied the pointwise strong consistency for a kind of recursive kernel estimator based WOD samples.

In this paper, we attempt to establish a Bernstein-type inequality and to derive the rates of strong convergence for the estimators of density and hazard rate functions for WOD samples. Throughout the paper, all limits are taken as *n* tends to ∞, and $c, C, C_{1}, C_{2}, \ldots $ denote positive constants whose values may change from one place to another, unless specified otherwise.

## Assumptions and some auxiliary results

For the sake of simplicity, some assumptions on kernel function $K(\cdot)$ and density function $f(x)$ are listed below.

1. $K(u)\in L_{1}$, $\int_{-\infty}^{+\infty} K(u)\,du =1$, $\sup_{x\in R}(1+|x|)|K(x)|\leq c<\infty$.
2. $\int_{-\infty}^{+\infty} u^{r}K(u)\,du =0$, $r=1,2,\ldots, s-1$, $\int_{-\infty}^{+\infty} u^{s} K(u)\,du =M\neq0$, where *M* is a positive constant, $s\geq2$ is some positive integer.
3. $f(x)\in C_{2,\alpha}$, where *α* is a positive constant, $C_{2,\alpha}$ implies that $f(x)$ is 2nd differentiable, $f^{\prime\prime}(x)$ is a continuous function, and $|f^{\prime \prime}(x)|\leq\alpha$.

The following proposition for WOD random sequence comes from Corollary 3 in Shen ([3], 2013), which will be used in the following.

### Lemma 2.1

(i) *Let a random sequence* $\{X_{n}, n\geq1 \}$ *be WOD with dominating coefficients* $g(n)$. *If* $\{ G_{n}(\cdot), n\geq1\}$ *is a nondecreasing* (*or nonincreasing*) *function sequence*, *then the random sequence* $\{ G_{n}(X_{n}), n\geq1\}$ *is still WOD with the same dominating coefficients* $g(n)$.
(ii) *Let a random sequence* $\{X_{n}, n\geq1 \}$ *be WOD*, *then for each* $n\geq1$ *and any* $s > 0$,
  $$E\exp\Biggl\{ s\sum_{i=1}^{n}X_{i} \Biggr\} \leq g(n)\prod_{i=1}^{n}E\exp \{sX_{i}\}. $$

### Remark 2.1

Condition (A1) is a reasonable condition, we can refer to condition (2) in Cai ([14], 1993) and condition (II) in Theorem S of Lin ([15], 1987). And by (A1), we can get the following lemma.

### Lemma 2.2

(see Lemma 3, [14], 1993, or [15], 1987)

*Let* $K(x)$ *be satisfied* (A1), *and* $f(\cdot) \in{L_{1}}$, *then*

(i) *for the continuous point of* $f(x)$,
  $$\begin{gathered} \lim_{h_{n} \to0} h_{n}^{-1} \int_{R} K\biggl(\frac{x - u}{h_{n}}\biggr)f(u)\,du = f(x),\\ \lim _{h_{n} \to0} h_{n}^{-1} \int_{R} K^{2}\biggl(\frac{x - u}{h_{n}}\biggr) f(u) \,du = f(x) \int_{R} {K^{2}} (u)\,du;\end{gathered} $$
(ii) *for all* $x,y \in R$, $x \ne y$,
  $$\lim_{h_{n} \to0} {h_{n}^{ - 1}} \int_{R} {K \biggl(\frac{{x - u}}{h_{n}} \biggr)} K \biggl( \frac{{y - u}}{h_{n}} \biggr)\,du = 0. $$

### Lemma 2.3

*By Lemma* 3.4 *of Li* ([16], 2017), *we obtain that*

$$h_{n}^{-2} \bigl\vert {E\hat{f}_{n}(x) - f(x)} \bigr\vert \le h_{n}^{-2} \biggl( \frac{h_{n}^{2}}{2} \biggl\vert { \int_{R} {K(u){f^{''}}(x - \xi{h_{n}}u){u^{2}} \, du} } \biggr\vert \biggr) \le C. $$

Now, we will establish a Bernstein-type inequality for a WOD random sequence as follows.

### Lemma 2.4

*Let a random sequence* $\{X_{n}, n\geq1 \}$ *be WOD with dominating coefficients* $g(n)$, *and* $EX_{i}=0$, $|X_{i}|\leq d_{i}$ *for* $i=1,\ldots, n$, *where* $\{d_{i}, 1\leq i\leq n\}$ *is a sequence of positive constants*. *For* $t>0$, *if* $t\cdot\max_{1\leq i \leq n} {d_{i}}\leq1$, *then for any* $\varepsilon>0$,

$$P \Biggl( \Biggl\vert \sum_{i=1}^{n}X_{i} \Biggr\vert >\varepsilon \Biggr)\leq2 g(n) \exp \Biggl\{ -t\varepsilon+t^{2} \sum_{i=1}^{n}EX_{i}^{2} \Biggr\} . $$

### Proof

By $1\leq i\leq n$, $|tX_{i}|\leq1 $ a.s. and noting that $1+x\leq e^{x}$ for $x\in R$, we have that

$$\begin{aligned} E \exp(t X_{i})&=\sum_{k=0}^{\infty} \frac{E (t X_{i})^{k}}{k!} \leq1+E (t X_{i})^{2} \biggl\{ \frac{1}{2!}+\frac{1}{3!}+\cdots \biggr\} \\ &\leq 1+t^{2}E X_{i}^{2}\leq\exp\bigl\{ t^{2} E X_{i}^{2}\bigr\} .\end{aligned} $$

For any $\varepsilon>0$, using Markov’s inequality and Lemma 2.1(ii), we can get

2.1 $$ \begin{aligned}[b] P\Biggl(\sum _{i=1}^{n}X_{i}>\varepsilon\Biggr)&\leq \exp{(-t\varepsilon)}E \exp \Biggl(t\sum_{i=1}^{n}X_{i} \Biggr) \leq g(n) \exp{(-t\varepsilon)}\prod_{i=1}^{n}E \exp(t X_{i}) \\ &\leq g(n) \exp \Biggl\{ -t\varepsilon+t^{2}\sum _{i=1}^{n} E X_{i}^{2} \Biggr\} . \end{aligned} $$

By Lemma 2.1(i), we see that the random sequence $\{ -X_{n},n\geq1\}$ is still WOD with dominating coefficients $g(n)$, then

2.2 $$ P \Biggl(\sum_{i=1}^{n}X_{i} \leq-\varepsilon \Biggr)=P \Biggl(\sum_{i=1}^{n}(-X_{i}) \geq\varepsilon \Biggr)\leq g(n) \exp \Biggl\{ -t\varepsilon+t^{2}\sum _{i=1}^{n} E X_{i}^{2} \Biggr\} . $$

Combining (2.1) and (2.2), we complete the proof of Lemma 2.4. □

### Corollary 2.1

*Let a random sequence* $\{X_{n}, n\geq1 \}$ *be WOD with dominating coefficients* $g(n)$, *and* $EX_{i}=0$, $|X_{i}|\leq d$, *a*.*s*. *for* $i=1,\ldots , n$, *where* *d* *is a positive constant*. *Set* $\sigma_{n}^{2}=n^{-1}\sum_{i=1}^{n}EX_{i}^{2}$, *then for any* $\varepsilon>0$,

$$P \Biggl(\frac{1}{n} \Biggl\vert \sum_{i=1}^{n}X_{i} \Biggr\vert >\varepsilon \Biggr) \leq2 g(n) \exp \biggl\{ -\frac{n\varepsilon^{2}}{2(2\sigma_{n}^{2}+ d \varepsilon)} \biggr\} . $$

### Proof

By Lemma 2.4, taking $t=\varepsilon/(2\sigma _{n}^{2}+d\varepsilon)$, we can get Corollary 2.1 immediately. □

### Remark 2.2

By comparison, the conditions of Lemma 2.4 are weaker than those of Theorem 4 in Shen ([17], 2013). So, Lemma 2.4 is a generalization of Theorem 4 in Shen ([17], 2013), and it extends the Bernstein-type inequality in Wang ([18], 2015, Lemma 2.2) from END to WOD sequence.

### Lemma 2.5

*Let a random sequence* $\{ X_{n}, n\geq1 \}$ *be WOD with dominating coefficients* $g(n)$. $F(x)$ *is a continuous distribution function*. *If there exists a positive constant* $\{ {{\tau_{n}}} \}$ *such that* ${\tau_{n}} \to0$ *and* $n{\tau_{n}^{2}}/[\log(n g^{3}(n))]\to\infty$, *then*

$$\sup_{x} \bigl\vert {{F_{n}}(x) - F(x)} \bigr\vert = o({\tau_{n}}),\quad \textit{a.s.} $$

*In particular*, *taking* $\tau_{n} = n^{-1/2} [\log(n g^{3}(n))(\log\log n)^{\delta}]^{1/2}$ *for some* $\delta>0$, *we have*

$$\sup_{x} \bigl\vert {{F_{n}}(x) - F(x)} \bigr\vert = o\bigl(n^{-1/2} \bigl[\log\bigl(n g^{3}(n)\bigr) (\log \log n)^{\delta}\bigr]^{1/2}\bigr), \quad\textit{a.s.} $$

### Proof

The proof is based on a modification of the proof of Lemma 3.5 in Li ([16], 2017). We only outline the difference. Let $x_{n,k}$ satisfy $F(x_{n,k})=k/n$, $k=1,2,\ldots,n-1$, and $\xi_{ik} = I(X_{i}\leq x_{n,k}) - EI(X_{i}\leq x_{n,k})$, then

2.3 $$ P \Bigl( \sup_{x\in R}\big| {{F_{n}}(x) - F(x)} \big| > \varepsilon{\tau _{n}} \Bigr)\le\sum_{k = 1}^{n - 1} P \Biggl(\frac{1}{n} \Biggl\vert \sum_{i = 1}^{n} \xi_{ik} \Biggr\vert >\frac{ \varepsilon\tau_{n}}{2} \Biggr). $$

By Lemma 2.1, it is easy to see that a random sequence $\{\xi _{ik}, i\geq1\}$ is WOD with $E\xi_{ik}=0$, $|\xi_{ik}| \le2$ for fixed *k*. Thus, by Lemma 2.4, and by choosing $t=\varepsilon \tau_{n}/4$ for *n* large enough, we have

2.4 $$ P \Biggl(\frac{1}{n} \Biggl\vert \sum_{i = 1}^{n} \xi_{ik} \Biggr\vert >\frac {\varepsilon\tau_{n}}{2} \Biggr) \le2 g(n)\exp \biggl\{ -\frac {\varepsilon^{2}n\tau_{n}^{2}}{16} \biggr\} \le2 g(n) \bigl[ n g^{3}(n) \bigr]^{-3}\leq2 n^{-3}. $$

By (2.3) and (2.4), we have

$$P \Bigl(\sup_{x\in R} \big| {{F_{n}}(x) - F(x)} \big| > \varepsilon{\tau _{n}} \Bigr) \le2\sum_{k= 1}^{n-1} n^{-3}\leq C_{3} n^{-2}. $$

Therefore, by the Borel–Cantelli lemma, we obtain the result of Lemma 2.5. □

## Main results and proofs

### Theorem 3.1

*Let a random sequence* $\{ X_{n}, n\geq1 \}$ *be WOD with dominating coefficients* $g(n)$, *and let* (A1)*–*(A3) *hold true*. *Let* $K(\cdot)$ *be a bounded monotonic density function*, *and* $n h_{n}^{6}/[\log(n g^{2}(n))(\log \log n)^{\delta}]\to0 $ *for some* $\delta> 0$. *Then*, *for* $f(x)\in C_{2,\alpha}$,

$$\bigl[ n h_{n}^{2}/\bigl[\log\bigl(n g^{2}(n) \bigr) (\log\log n)^{\delta}\bigr] \bigr]^{1/2}\bigl( \hat{f}_{n}(x) - f(x)\bigr) \to0,\quad \textit{a.s.} $$

Weakening the condition of density kernel $K(\cdot)$ from bounded monotonic density function to bounded variation function, we can get the result as follows.

### Corollary 3.1

*Let a random sequence* $\{ X_{n}, n\geq1 \}$ *be WOD with dominating coefficients* $g(n)$, *and let* (A1)*–*(A3) *hold true*. *If the density kernel* $K(\cdot)$ *is a Borel measurable and bounded variation function and* $nh_{n}^{6}/[\log(n g^{2}(n))(\log\log n)^{\delta}]\to0 $ *for some* $\delta> 0$, *then*, *for* $f(x)\in C_{2,\alpha}$,

$$\bigl[n h_{n}^{2}/\bigl[\log\bigl(n g^{2}(n)\bigr) (\log\log n)^{\delta}\bigr] \bigr]^{1/2}\bigl( \hat{f}_{n}(x) - f(x)\bigr) \to0, \quad\textit{a.s.} $$

### Theorem 3.2

*If*, *in addition to the assumptions of Theorem *3.1 *and Lemma *2.5, *the distribution function* $F({x_{0}}) < 1$, *then for* $x \le{x_{0}}$,

$$\bigl[ n h_{n}^{2} /\bigl[\log\bigl(n g^{2}(n) \bigr) (\log\log n)^{\delta}\bigr] \bigr]^{1/2}\bigl(\hat{ \lambda}_{n}(x) - \lambda(x)\bigr) \to0, \quad\textit{a.s.} $$

### Corollary 3.2

*If*, *in addition to the assumptions of Corollary *3.1 *and Lemma *2.5, *the distribution function* $F({x_{0}}) < 1$, *then for* $x \le{x_{0}}$,

$$\bigl[ n h_{n}^{2}/\bigl[\log\bigl(n g^{2}(n) \bigr) (\log\log n)^{\delta}\bigr] \bigr]^{1/2}\bigl(\hat{ \lambda}_{n}(x) - \lambda(x)\bigr) \to0, \quad\textit{a.s.} $$

### Remark 3.1

From Theorems 3.1 and 3.2, Corollaries 3.1 and 3.2, the rates of strong convergence can nearly reach $O(n^{-2/5})$ by choosing bandwidth $h_{n}=O(n^{-1/5})$. So the rates in here reach the same order of convergence as those in Li ([16], 2017) and Li and Yang ([19], 2005). Note that negative association implies WOD, and END also implies WOD, but the converse does not hold. Then the results of this paper are the generalization of those in Li ([16], 2017) and Li and Yang ([19], 2005).

### Proof of Theorem 3.1

Writing

3.1 $$ n{h_{n}}\bigl[\hat{f}_{n}(x) - E\hat{f}_{n}(x) \bigr] = \sum_{j = 1}^{n} \biggl[ K \biggl( \frac{x - X_{j}}{h_{n}} \biggr) - EK \biggl(\frac{x-X_{j}}{h_{n}} \biggr) \biggr] = \sum _{j= 1}^{n} Y_{j}, $$

where

$$Y_{j} = K \biggl(\frac{x - X_{j}}{h_{n}} \biggr) - EK \biggl( \frac {x-X_{j}}{h_{n}} \biggr). $$

By Lemma 2.1, we see that a random sequence $\{ Y_{j}, 1\leq j\leq n \}$ is still WOD with dominating coefficients $g(n)$. And by $K(x)$ is bounded, we can get that $EY_{j}=0$, $|Y_{j}| \le C_{3}$,

$$EY_{j}^{2} = EK \biggl(\frac{x - X_{j}}{h_{n}} \biggr)^{2} \le C_{3}\quad \hbox{and} \quad\sigma_{n}^{2}=n^{-1} \sum_{j= 1}^{n} EY_{j}^{2} \leq C_{3}. $$

Set $\theta_{n} = \{ nh_{n}^{2}/ [\log(n g^{2}(n))(\log\log n)^{\delta}] \} ^{-1/2}$, by Corollary 2.1, for any $\varepsilon>0$,

$$\begin{gathered} P \Biggl(\frac{1}{n} \Biggl\vert \sum_{j= 1}^{n} Y_{j} \Biggr\vert > h_{n} \theta _{n}\varepsilon \Biggr) \\ \quad\le 2g(n)\exp \biggl\{ - \frac{n(\theta_{n} h_{n}\varepsilon)^{2}}{2[2\sigma _{n}^{2}+ C_{3}(\theta_{n} h_{n} \varepsilon)]} \biggr\} \\ \quad \le2g(n)\exp \biggl\{ - \frac{\log(n g^{2}(n))(\log\log n)^{\delta}\varepsilon^{2}}{2 \{2C_{3} +n^{-1/2}[\log(n g^{2}(n))(\log\log n)^{\delta}]^{1/2} C_{3} \varepsilon \}} \biggr\} \\ \quad\le2g(n) \bigl(n g^{2}(n)\bigr)^{-2}=2n^{-2}.\end{gathered} $$

Thus, by the Borel–Cantelli lemma, we have

3.2 $$ \frac{1}{nh_{n}\theta_{n}} \Biggl\vert \sum_{j= 1}^{n} Y_{j} \Biggr\vert \to0, \quad\text{a.s.} $$

Hence, using (3.1) and (3.2), we get

3.3 $$ \theta_{n}^{-1}\bigl[\hat{f}_{n}(x) - E \hat{f}_{n}(x)\bigr] \to0,\quad \text{a.s.} $$

Note that

3.4 $$ \theta_{n}^{-1}\bigl[\hat{f}_{n}(x) - f(x)\bigr]= \theta _{n}^{-1}\bigl[\hat{f}_{n}(x) - E \hat{f}_{n}(x)\bigr]+\theta_{n}^{-1}\bigl[E \hat{f}_{n}(x) - f(x)\bigr] $$

and

$$\theta_{n}^{-1} h_{n}^{2}= \frac{\sqrt{nh_{n}^{6}}}{\sqrt{ \log(n g^{2}(n))(\log\log n)^{\delta}}} \to0. $$

Then, by Lemma 2.3, we have

3.5 $$ \theta_{n}^{-1}\bigl[E\hat{f}_{n}(x) - f(x)\bigr] = \frac {h_{n}^{2}}{\theta_{n}}\cdot\frac{1}{h_{n}^{2}}\cdot\bigl[E\hat{f}_{n}(x) - f(x)\bigr] \to0. $$

It follows by using (3.3)–(3.5) that

$$\bigl\{ nh_{n}^{2}/ \bigl[\log\bigl(n g^{2}(n) \bigr) (\log\log n)^{\delta}\bigr] \bigr\} ^{1/2}\bigl[ \hat{f}_{n}(x) - f(x)\bigr] \to0,\quad \text{a.s.} $$

This completes the proof of Theorem 3.1. □

### Proof of Corollary 3.1

By $K(x)$ is a bounded variation function, we can write that $K(x)=K_{1}(x)-K_{2}(x)$, where $K_{1}(x)$ and $K_{2}(x)$ are two monotone increasing functions. Then

3.6 $$ n{h_{n}}\bigl[\hat{f}_{n}(x)- E\hat{f}_{n}(x)\bigr] = \sum_{j= 1}^{n} Y_{1j}-\sum _{j= 1}^{n} Y_{2j}, $$

where

$$Y_{ij} = K_{i} \biggl(\frac{x - X_{j}}{h_{n}} \biggr)-EK_{i} \biggl(\frac{x - X_{j}}{h_{n}} \biggr) \quad\hbox{for } i=1,2. $$

Then the following proof of Corollary 3.1 is the same as the proof of Theorem 3.1, here it is omitted. □

### Proof of Theorem 3.2

Set $S(x)=1-F(x)$, $S_{n}(x)=1-F_{n}(x)$. By the hazard rate estimator (1.2), we get

3.7 $$ \bigl\vert \hat{\lambda}_{n}(x) - \lambda(x) \bigr\vert \le \frac{{S(x) \vert {\hat{f}_{n}(x) - f(x)} \vert + f(x) \vert S_{n} (x) - S (x) \vert }}{{S (x)S_{n} (x)}}. $$

Note that $0 < S(x_{0}) \le S(x) \le1$ for $x \le x_{0}$, and $\sup_{x} f(x) \le C_{5}$. It follows by Theorem 3.1 and Lemma 2.5 that

3.8 $$ \bigl[nh_{n}^{2}/\bigl[\log\bigl(n g^{2}(n)\bigr) (\log\log n)^{\delta}\bigr] \bigr]^{1/2}\bigl( \hat{f}_{n}(x) - f(x)\bigr) \to0,\quad \text{a.s.} $$

and

3.9 $$ n^{1/2}/\bigl[(\log\bigl(n g^{3}(n)\bigr) (\log\log n)^{\delta }\bigr]^{1/2}\sup_{x \le x_{0}} \bigl\vert S_{n}(x) - S(x) \bigr\vert \to0,\quad \text{a.s.} $$

On the other hand, for *n* large enough, as $x \le x_{0}$, we have

$$S_{n}(x) > S(x) - S(x_{0}) >\frac{1}{2} S(x_{0}) > 0. $$

Hence, from (3.7), (3.8), and (3.9), we have

$$\bigl[nh_{n}^{2}/\bigl[\log\bigl(n g^{2}(n)\bigr) (\log\log n)^{\delta}\bigr] \bigr]^{1/2}\bigl(\hat{ \lambda}_{n}(x) - \lambda(x)\bigr) \to0,\quad \text{a.s.} $$

Then we obtain Theorem 3.2. □

### Proof of Corollary 3.2

The proof is analogous to the one of Theorem 3.2 by Corollary 3.1, and here it is also omitted. □

## Conclusion

In Sect. 2, we give a Bernstein-type inequality for WOD random sequence which extends the Bernstein-type inequality based END sequence. In Sect. 3, using the Bernstein-type inequality, we obtain the rates of strong convergence for the estimators of density and hazard rate functions. By choosing the bandwidth $h_{n}=O(n^{-1/6})$, the rates of strong convergence can nearly reach $O(n^{-1/3})$.
